# Health situation of adults in Germany – Results for selected indicators from GEDA 2019/2020-EHIS

**DOI:** 10.25646/8459

**Published:** 2021-09-15

**Authors:** Christin Heidemann, Christa Scheidt-Nave, Ann-Kristin Beyer, Jens Baumert, Roma Thamm, Birga Maier, Hannelore Neuhauser, Judith Fuchs, Ronny Kuhnert, Ulfert Hapke

**Affiliations:** Robert Koch Institute, Berlin Department of Epidemiology and Health Monitoring

**Keywords:** SUBJECTIVE HEALTH, DEPRESSIVE SYMPTOMS, CHRONIC DISEASES, HEALTH MONITORING

## Abstract

In this article, we examine selected health indicators for the adult population aged 18 years and older in Germany (n=22,708) from the German Health Update (GEDA 2019/2020-EHIS) conducted between April 2019 and September 2020. These indicators include those of self-assessed health and depressive symptoms as well as chronic physical diseases and conditions. In young adulthood (18 to 44 years), over 80% of participants report good or very good subjective health. During this phase of life, most chronic diseases and conditions are rare, although allergies are frequent, and bronchial asthma and depressive symptoms are not uncommon. From mid adulthood (45 years and older), there is a gradual increase in the prevalence of chronic diseases such as cardiovascular disease, diabetes, chronic obstructive pulmonary disease and osteoarthritis. Over 60% of older adults (65 years and older) report a chronic disease or long-term health problem, while only half continue to report good or very good subjective health. During this stage of life, allergies and depressive symptoms become less prevalent. For some diseases, there are also differences according to gender and level of education. This article demonstrates the high public health relevance of age-associated chronic physical diseases and health related limitations in everyday life in an ageing society as well as the need to provide care for certain health conditions already in young adulthood.

## 1. Introduction

As a population-representative health survey of the adult population in Germany, the German Health Update (GEDA) is an important component of continuous health monitoring at the Robert Koch Institute (RKI) [1]. Since 2014/2015, the questionnaire of the European Health Interview Survey (EHIS), which is conducted every five years to assess the health situation of the population aged 15 years and older, has been incorporated into GEDA [2]. The GEDA part on health problems and diseases focuses on self-assessed general health, health disorders and the resulting limitations in everyday life as well as mental health and common non-communicable diseases. The reason for this focus lies in the fact that chronic conditions and non-communicable diseases and their risk factors dominate morbidity and mortality in European countries and interact with the persistent threat from infectious diseases [3]. For example, the widespread prevalence of non-communicable diseases, multimorbidity and frailty in the population has contributed to the severe health consequences of the current global COVID-19 pandemic [4, 5]. Conversely, we cannot rule out the possibility that chronic health issues will increase at the population level in the context of the pandemic. These could include complications following infection with SARS-CoV-2 and indirect health consequences of the pandemic caused by restricted social contact and by other non-pharmaceutical measures of infection control [6–8]. This reinforces the demands of international health organisations for targeted health surveillance to prevent and control non-communicable diseases and to promote physical and mental health on a national and global level [9, 10].

**GEDA 2019/2020-EHIS**

Fifth follow-up survey of the German Health Update

**Data holder:** Robert Koch Institute

**Objectives:** Provision of reliable information on the health status, health behaviour and health care of the population living in Germany, with the possibility of European comparisons

**Study design:** Cross-sectional telephone survey

**Population:** German-speaking population aged 15 and older living in private households that can be reached via landline or mobile phone

**Sampling:** Random sample of landline and mobile telephone numbers (dual-frame method) from the ADM sampling system (Arbeitskreis Deutscher Markt- und Sozialforschungsinstitute e.V.)

**Sample size:** 23,001 respondents

**Study period:** April 2019 to September 2020

**GEDA survey waves:**

▶ GEDA 2009
▶ GEDA 2010
▶ GEDA 2012
▶ GEDA 2014/2015-EHIS
▶ GEDA 2019/2020-EHIS

Further information in German is available at www.geda-studie.de

The standardised EHIS questionnaire, approved at European level, comprises four modules for collecting data on health status, health determinants, health care and sociodemographic background [2]. The Minimum European Health Module (MEHM) consists of three main health status indicators: self-assessment of general health (subjective health), presence of chronic diseases or long-term health problems, and presence of health-related limitations in usual everyday activities [11]. The information collected in GEDA can be used to monitor further indicators, including individual chronic diseases, accidents and injuries, depressive symptoms and functional aspects of health such as pain, restrictions to mobility and the need for help in everyday life [2].

Based on data from GEDA 2019/2020-EHIS, this article provides an initial overview of the current health of adults in Germany using selected indicators. The data collection was conducted between April 2019 and September 2020 and thus includes the period of severe contact restrictions imposed in response to the COVID-19 pandemic between mid-March and early June 2020. However, this article aims to assess the health situation over the entire survey period. Findings on the above-mentioned indicators of health status, chronic non-communicable diseases of particular public health relevance and depressive symptomatology as an indicator of mental health are differentiated by age group and gender with the goal of mapping health within different phases of adulthood. Differences in education are reported in relation to health inequalities.

## 2. Methodology

### 2.1 Study design and sample

The German Health Update (GEDA) is a cross-sectional survey based on a nationwide sample of the resident population in Germany. The GEDA study has been conducted by the Robert Koch Institute (RKI) on behalf of the German Federal Ministry of Health at multi-year intervals since 2008 and is part of the health monitoring at the RKI [1, 12]. The fifth follow-up survey, GEDA 2019/2020-EHIS, took place between April 2019 and September 2020. As in the 2014/2015 wave, the questionnaire of the European Health Interview Survey (EHIS) was fully integrated [2, 13]. GEDA 2019/2020-EHIS was conducted as a telephone interview survey using a computer assisted, fully structured interview (i.e. Computer Assisted Telephone Interview, CATI). It was based on a random sample of landline and mobile telephone numbers (dual-frame method) [14]. The target population comprised the population aged 15 years and older living in private households and with permanent residency in Germany. A total of 23,001 people provided complete interviews. GEDA 2019/2020-EHIS used gender identities to describe gender differences and allowed the respondents to indicate which gender they feel they belong to. Respondents 15 years and older included 12,101 women and 10,838 men. 62 respondents provided a different gender identity to the one that they were assigned at birth or gave no information. These individuals are not included in the gender stratified analyses. Based on the standards of the American Association for Public Opinion Research (AAPOR), the response rate was 21.6% (RR3) [15]. A detailed description of the methodology used for GEDA 2019/2020-EHIS, including a differentiated presentation of the response rates, can be found in Allen et al. in this issue of the Journal of Health Monitoring [16].

### 2.2 Indicators

#### Self-assessed health status

Data on three health status indicators were collected as part of the MEHM and as a central component of all national health surveys in the European Union [11, 17]. The indicator for subjective health is measured with the following question on self-assessed general health, as recommended by the World Health Organization (WHO): ‘How is your health in general?’. Participants were asked to choose one of five response options: ‘very good’, ‘good’, ‘fair’, ‘bad’ or ‘very bad’. The nationwide health monitoring defines the answers ‘very good’ or ‘good’ as a positively perceived subjective health. The indicator for a chronic disease or long-term health problem was collected via the following question: ‘Do you have any chronic disease or a long-term health problem? This means diseases or health problems that have lasted or are expected to last for at least 6 months’. The response options were ‘yes’, ‘no’ or ‘don’t know’. The indicator for health-related limitations in usual everyday activities (Global Activity Limitation Indicator, GALI) was measured using a two-stage approach. The initial question was: ‘Are you limited by a health problem in activities of your normal everyday life? Would you say you are…’ with the response options being ‘… severely limited’, ‘… moderately limited’ and ‘… not limited’. Respondents who gave one of the first two response options were asked further: ‘How long have you been limited?’. Response options were ‘less than 6 months’ and ‘6 months or longer’.

#### Depressive symptoms

The presence of depressive symptoms in the past two weeks was used as an indicator of mental health and was recorded via participants’ self-assessment using the internationally established 8-item Patient Health Questionnaire (PHQ-8) [18]. With this questionnaire, the symptoms of major depression occurring in the past two weeks are rated according to the Diagnostic and Statistical Manual of Mental Disorders (DSM-IV, 4th edition [19]) as ‘not at all’, ‘several days’, ‘more than half of the days’ or ‘nearly every day’. The presence of depressive symptoms is assumed from a sum score of at least ten of the maximum of 24 points.

#### Chronic physical diseases and health conditions

Data on the 12-month prevalence of chronic diseases and health problems are based on responses to the following question: ‘This section deals with lasting diseases and chronic health problems. Please do not include temporary health problems. In the past 12 months, have you had any of the following diseases or health problems?’. A list included in the questionnaire asked specific questions about individual diseases and complaints, with possible answers being ‘yes’, ‘no’ or ‘don’t know’. This article considers the information collected on diabetes mellitus (queried as ‘diabetes, not gestational diabetes’), coronary heart disease (CHD, queried as ‘heart attack’, ‘chronic consequences of heart attack’ and ‘coronary heart disease or angina pectoris’), stroke or chronic consequences of stroke (queried as ‘stroke’ and ‘chronic consequences of stroke’), chronic obstructive pulmonary disease (COPD, queried as ‘chronic bronchitis, chronic obstructive pulmonary disease, emphysema’), bronchial asthma (queried as ‘asthma, including allergic asthma’), allergies (queried as ‘allergies such as hay fever, allergic reactions of the eyes or skin, food allergies or other allergies, not including allergic asthma’) and osteoarthritis (queried as ‘osteoarthritis, not including rheumatoid arthritis or joint inflammation’).

### 2.3 Statistical analyses

The analyses are based on data from 22,708 participants aged between 18 and 99 years (11,959 women, 10,687 men, and 62 participants who reported a different or no gender identity). For each indicator, participants who did not provide information for the variables on which a specific indicator is based were excluded from the analyses (12 for subjective health, 69 for chronic disease/long-term health problem, 57 for long-term health-related limitation in everyday activities, 447 for depressive symptoms, 34 for diabetes, 122 for CHD, 16 for stroke/chronic consequence of stroke, 26 for bronchial asthma, 42 for COPD, 85 for allergies and 159 for osteoarthritis). The results are presented as prevalence in percentages with a 95% confidence interval (95% CI) for women and men separated by age group (18- to 29-year-olds, 30- to 44-year-olds, 45- to 64-year-olds, 65- to 79-year-olds, and at least 80 year-olds) and according to education level (International Standard Classification of Education, ISCED: low, medium and high education group).

The analyses were carried out using a weighting factor to correct for deviations of the sample from the population structure. Design weighting was first carried out for the different selection probabilities (mobile and landline). This was followed by an adjustment to the official population figures based on age, sex, federal state and district type (as of 31 December 2019). Adjustments were also undertaken to ensure the data reflected the education distribution identified by the 2017 microcensus. This was conducted in accordance with ISCED classifications [20].

The analyses were carried out with SAS 9.4. In order to take the weighting appropriately into account when calculating the confidence intervals and p-values, all analyses were calculated using the SAS survey procedures. A statistically significant difference between groups is assumed if the corresponding p-value in the Rao-Scott Chi-Square test is less than 0.05.

## 3. Results

### 3.1 Self-assessed health status

Overall, 69.9% (95% CI 69.0%–70.9%) of participants rated their subjective health as very good or good, with the proportion of women (68.6%) being slightly lower than that of men (71.6%). Both genders perceived their health considerably poorer with increasing age (Table 1). Thus, in the youngest age group (18 to 29 years), 87.2% of women and 88.3% of men regarded their health as very good or good compared to 42.5% of women and 52.6% of men in the oldest age group (80 years and older).

49.2% (95% CI 48.2%–50.2%) of the participants reported a chronic disease or a long-term health problem; this proportion was slightly higher for women (51.9%) than for men (46.4%). The corresponding proportion increased with increasing age from 33.8% for women and 25.8% for men in the youngest age group to 61.9% for women and 62.0% for men in the oldest age group.

Long-term health-related limitations in usual everyday activities were reported by 33.4% (95% CI 32.4%–34.4%) of the participants. The prevalence was higher for women (35.5%) than for men (31.0%). A substantial increase in the prevalence of health-related limitations can be seen with increasing age, from 16.8% for women and 10.5% for men in the youngest age group to 63.2% for women and 58.1% for men in the oldest age group.

For all three indicators, there is a pronounced educational gradient, particularly for women, with a lower prevalence of very good or good subjective health and a higher prevalence of chronic diseases or long-term health problems as well as of long-term health-related limitations in everyday life in the low education group compared to the high education group. Such a pattern largely persists across the age groups (Annex Table 1).

### 3.2 Depressive symptoms

A total of 8.3% (95% CI 7.7%–9.0%) of adults reported depressive symptoms within the previous two weeks. 8.8% of women and 7.5% of men are affected (Table 2). In the youngest adult age group (up to 29 years), more women tend to be affected. Depressive symptoms were least likely to be reported in the 65- to 79-year-old age group. For both women and men, the frequency of depressive symptoms decreases with higher levels of education. Compared to the high education group, almost three times as many women in the lower education group and four times as many men are affected by depressive symptoms. An analysis of depressive symptoms by age and education group (Annex Table 2) shows that the differences between the genders diminish with increasing age and higher education.

### 3.3 Chronic physical diseases and health conditions

#### Cardiometabolic diseases

Overall, 8.9% (95% CI 8.4%–9.5%) of adults reported the presence of diabetes mellitus (excluding gestational diabetes) in the past twelve months, with the prevalence for women (8.2%) being lower than for men (9.6%) (Table 3). In young adulthood (up to 44 years of age), the prevalence for both women and men is still below 3.5%, but then rises with age, reaching 17.9% in women and 22.3% in men in the oldest age group.

A total of 5.8% (95% CI 5.4%–6.3%) of adults reported CHD (i.e. heart attack, chronic consequences of a heart attack, coronary heart disease or angina pectoris) in the past twelve months, with the prevalence in women (5.1%) also lower than in men (6.6%). Cases of CHD are rare in young adulthood for both genders (less than 1%) and rise with age to 18.9% in women and 21.9% in men in the oldest age group.

A total of 2.3% (95% CI 2.0%–2.6%) of adults reported a stroke or chronic consequences of stroke in the past twelve months, with women (2.1%) and men (2.3%) showing similar prevalence estimates. In young adulthood, prevalence is still below 1% for both genders and then rises to 5.5% in women in the age group 80 years and older and to 6.2% in men aged 65 to 79 years.

For the cardiometabolic diseases under consideration, a clear educational gradient can be observed in women, with prevalence estimates around twice as high in the medium education group and around three times as high in the lower education group compared to the higher education group. In men, the lowest prevalence estimates are also found in the high education group, but the differences of the high education group with the medium and low education groups are much less pronounced and, in some cases, not statistically significant.

#### Diseases of the lower respiratory tract

A total of 6.1% (95% CI 5.6%–6.7%) of adults reported the presence of COPD in the past twelve months. Prevalences for women (6.5%) and men (5.8%) are similar (Table 4). COPD prevalence increases with age from less than 2% in both genders for the 18- to 29-year age group to 10.9% for women in the 80 years and older age group and 10.4% for men in the 65- to 79-year age group. Considerable differences regarding levels of education can be observed for both genders, with higher prevalences in the low and medium education groups compared to the high education group.

The prevalence of bronchial asthma (including allergic asthma) in the past twelve months for adults was 8.0% (95% CI 7.5%–8.6%), with women (9.1%) more frequently affected than men (7.0%). Prevalence estimates are similar for women and men across all age groups and no statistically significant differences by education group are evident.

#### Allergies

The presence of (any) allergy (except allergic asthma) in the past twelve months was reported by almost one-third of adults (30.9%, 95% CI 30.0%–31.8%), with women (34.7%) being affected more often than men (27.0%) (Table 5). An allergy was mainly reported in young and mid-adulthood (women up to 64 years, men up to 44 years). In addition, women in the high education group more frequently reported an allergy than women in the low education group.

#### Osteoarthritis

A total of 17.1% (95% CI 16.4%–17.8%) of adults reported having osteoarthritis in the past twelve months, with women (21.6%) being affected markedly more often than men (12.4%) (Table 6). For both genders, prevalence does not substantially exceed 5% in young adulthood, but then increases with age to 47.3% in the oldest women and 31.6% in the oldest men. While a clear educational gradient is evident for women with the lowest prevalence found in the high education group and the highest prevalence in the low education group, no such gradient is found for men.

## 4. Discussion

In this article, we present current data on selected indicators of physical and mental health among adults in Germany, which are collected every five years as part of EHIS integrated into the nationwide GEDA study.

The results are differentiated according to five age groups, each representing a phase of life, and by gender. From the age of 45 years, the prevalence of subjective health assessed as good or very good declines substantially to 43% for women and 53% for men, and health-related limitations in usual everyday activities lasting at least six months rise considerably to 63% for women and 58% for men. The prevalence of having (any) chronic disease or long-term health problem for at least six months increases gradually with age in both genders. Among the individual chronic diseases examined, CHD, diabetes, COPD and osteoarthritis characteristically increase in middle age (45 years and older) for both genders, with 12-month prevalences in the oldest age group for both women and men of around 6% for stroke, around 20% for diabetes and CHD, around 10% for COPD and for osteoarthritis, 47% in women and 32% in men. In contrast, depressive symptoms in the previous two weeks are especially common in young and mid-adulthood with prevalences between 7% and 12% for both genders. A similar picture emerges for allergies, which, with a 12-month prevalence of around 40%, are most frequently reported by women in young and mid-adulthood and by men in young adulthood. Only bronchial asthma exhibits no significant differences in 12-month prevalence across the age range for both genders.

With the exception of bronchial asthma and allergies, all the health indicators considered show an educational gradient to the disadvantage of adults with lower levels of education. For most of the indicators, this is particularly pronounced in women, and for osteoarthritis, it only affects women. Conversely, a higher prevalence of allergies is observed in women with a high level of education.

### 4.1 Self-assessed health status

Subjective health primarily reflects how well a person feels. A less favourable self-assessment of health is associated with a higher prevalence of chronic diseases and health problems [21–23] and is also an important predictor of premature mortality [24]. Based on the present study, around 70% of all adults in Germany rate their subjective health as very good or good; about half report a chronic disease or a long-term health problem and a third report severe or moderate long-term health-related limitations in usual everyday activities, each of which have lasted at least six months. Over the course of life, subjective health is assessed more negatively as age increases, and the presence of chronic diseases or health problems as well as of health-related limitations are correspondingly reported more frequently. This pattern over the course of life points to the association between self-assessed subjective health and the actual state of health. Said differently, the more frequently people report chronic diseases, health problems or health-related limitations, the more negatively they assess their state of health; a finding that is also in line with other studies [21–23]. In comparison to earlier RKI surveys, the proportions of women and men with very good or good subjective health were similar in the three telephone surveys GEDA 2009 to 2012 and somewhat lower in GEDA 2014/2015-EHIS, which collected data in writing or online [25]. The collection of data for the indicators on chronic diseases and long-term health problems as well as on long-term health-related limitations in usual everyday activities differed in previous GEDA waves to the one used here, which limits the possibilities for comparisons over time. In the present study, gender-, age- and education-specific differences in the prevalences of all three indicators were observed, which could enable approaches for the improvement of target group-specific prevention measures as well as health promotion and health care.

### 4.2 Depressive symptoms

Depressive symptoms not only occur with depression but can also be accompanying or secondary symptoms of other mental disorders or physical diseases or refer to sub-clinical forms of depression. It should therefore be noted that the PHQ-8 questionnaire-based indicator for depressive symptoms correlates with almost all domains of mental health and covers a total of eight symptom domains. For reasons of space, however, this article only presents the total score. Especially in young adulthood, women are more frequently affected by depressive symptoms. Earlier analyses of time trends have shown that there can be considerable changes within the different age groups. An analysis on major depression, for example, showed that the prevalence of depression among 18- to 34-year-old women almost doubled from 8.8% to 15.6% between 1998 and 2011, while the prevalence decreased from 9.8% to 5.0% among 50- to 65-year-old women [26]. Analyses of GEDA 2014/2015-EHIS data also showed that younger women are affected by depressive symptoms more frequently than older women [27]. The current analyses of self-reported depressive symptoms in the past two weeks replicate this finding, indicating entrenched risks for younger women. No obvious differences were observed for men in the age groups up to 64 years, as was also the case in GEDA 2014/2015-EHIS. Only in the age group 65 years and older does prevalence decrease, as it also does for women. Differences in the prevalence of depressive symptoms according to levels of education have tended to increase rather than decrease. Further in-depth trend analyses would be needed to determine whether this development is due to a worsening of the situation for people in the low education group or an improvement in the situation for higher education groups. The complexity of the possible backgrounds and causal relationships is discussed, for example, in the current Health Situation of Women in Germany Report [28] and the Focus Report on Mental Health [29] of the RKI. In any case, this is a possible indication that current preventive approaches, such as the expansion of the Occupational Health and Safety Act through risk assessment (sub-para. 6 in §5 of the Occupational Health and Safety Act, ArbSchG) should be reviewed to see how effectively they also reach the population with lower levels of education.

### 4.3 Chronic physical diseases and health conditions

#### Cardiometabolic diseases

The metabolic disease diabetes mellitus, which is characterised by a disturbance of blood sugar level regulation, plays an important role from mid-adulthood onwards. Thus, data on 12-month prevalence show that in the age range 45 to 64 years almost one in ten people and from 65 years even one in five people report a diabetes. Overall, women are affected less frequently than men, and the low and medium education groups are affected more often than the high education group. These age, gender and education-specific differences have also been observed in previous studies [30–32]. Beyond the age of 45 years, diabetes usually develops as type 2 diabetes. Gestational diabetes, which becomes relevant for women in young adulthood, was explicitly excluded from the question in GEDA 2019/2020-EHIS. Also disregarded in the present study are undiagnosed cases of diabetes, which contribute around 2% to the overall prevalence of diabetes in the adult population [30]. While the prevalence of undiagnosed diabetes has decreased over the past few decades, the prevalence of diagnosed diabetes has increased [33]. The current prevalence estimate is also slightly higher than that reported by GEDA 2014/2015-EHIS [31]. This may be due to various factors, such as an earlier diagnosis of diabetes, improved care for diagnosed cases and the demographic ageing of the population [33]. As described in the context of the diabetes surveillance in Germany established at the RKI, diabetes and its concomitant and secondary diseases adversely impact quality of life, reduce a person’s healthy life years and lower life expectancy [34, 35]. For this reason, in addition to optimal medical care oriented to the needs of those affected, increased primary preventive behavioural and settings-based measures are necessary to prevent the development of diabetes and consequently to reduce the prevalence of diabetes in the population.

With almost three million cases, diseases of the circulatory system were the most common reason for hospital-isation in 2019 and with over 330,000 deaths, they were also the most common cause of death. Cardiovascular diseases were not surveyed comprehensively in the context of GEDA 2019/2020-EHIS, but only on the basis of the defined EHIS indicators. The 12-month CHD prevalence of 5.1% for women and 6.6% for men described here differ slightly from the age-standardised CHD prevalence of 3.9% for women and 8.0% for men calculated on the basis of the 2018 ambulatory claims data [36]. This difference could be caused by the relatively low case number for men from the low education group in GEDA, which may have led to an underestimation of CHD prevalence in men. This small number of cases may also have contributed to the study result that the known social status gradient for CHD is not pronounced among men, although it is clearly evident in women [37]. As both the cited study and GEDA data show, men are more likely to develop CHD than women. This has been observed in many studies [38]. In GEDA 2019/2020-EHIS, 2.1% of women and 2.3% of men answered yes to the question of whether they had had a stroke or chronic consequences of stroke in the past twelve months. These prevalence estimates were slightly lower in GEDA 2014/2015-EHIS [39], but due to the methodological differences between the two surveys, comparisons should be interpreted with caution. Furthermore, as expected, the 12-month prevalence is lower than the lifetime prevalence of stroke in 40- to 79-year-old women (2.5%) and men (3.3%), which was surveyed in the German Health Interview and Examination Survey for Adults (DEGS1, 2008–2011) [40]. As in previous surveys, there is also an age and education gradient for stroke [39] that is less pronounced than in CHD. Limiting factors here are the low participation rate for men with low levels of education and the question of whether the EHIS indicator is suitable for recording stroke prevalence in the population in a European comparison, as already discussed in regard to GEDA 2014/2015-EHIS [39]. Data on cardiovascular diseases, such as those collected here, help to determine the extent of the diseases within the population, to plan targeted prevention and care services and to monitor their effects.

#### Diseases of the lower respiratory tract

Irreversible and chronically progressive damage to the lung tissue in COPD leads to a permanent narrowing of the airways, overinflation of the lungs and obstruction of gas exchange, resulting in shortness of breath. Because COPD is difficult to assess, GEDA uses a variety of terms in its question (chronic bronchitis, chronic obstructive pulmonary disease, emphysema) in accordance with international epidemiological studies [41]. It should be noted that self-reports lead to considerably lower prevalence estimates of COPD than those based on pulmonary function tests, which can detect early stages [41]. The indicator used in GEDA 2019/2020-EHIS was also used in GEDA 2014/2015-EHIS. Although a direct comparison between the two survey waves is limited, mainly due to changes in sampling design, the results are very similar, particularly for men, and show 12-month prevalence estimates rising from the age of 45 years for both genders [41]. As expected, there are educational differences in the prevalence of COPD for both genders, reflecting inequalities in terms of the major risk factors (i.e. tobacco and pollutant exposure). For women, the prevalence has increased in comparison to GEDA 2014/2015-EHIS [41]. This most likely reflects gender-related changes in smoking behaviour, with a decrease in the proportion of male smokers and a further long-term increase in the proportion of female smokers. A convergence of COPD mortality rates in women and men, as well as of incidence and mortality rates from malignant tumours of the lungs, bronchi and trachea, has been observed for some time [42]. COPD is one of the leading causes of premature mortality, diminished quality of life and health-related impairment when performing everyday activities [41]. At an epidemiological level, this means that continuous health monitoring and embedding COPD in NCD surveillance are central building blocks for the promotion of public health.

Bronchial asthma is a chronic respiratory disease characterised by symptoms such as wheezing, shortness of breath and breathlessness, as well as a feeling of tightness in the chest or coughing. Similar to allergies, a number of disease mechanisms play a role in asthma, and there are allergic and non-allergic forms [43]. The current study indicates no variation by age in the overall 12-month prevalence of asthma in adults (8%), yet there is a gender difference already documented in numerous epidemiological studies, with women affected more frequently (9% versus 7% in men). In addition to this gender difference, the present study shows a known tendency toward higher prevalence estimates in lower education groups, although this is not statistically significant. The prevalence of asthma in GEDA 2014/2015-EHIS was slightly lower [44]. Bronchial asthma is one of the most common chronic diseases worldwide. Increasing numbers of people are affected, necessitating further efforts in prevention, diagnosis and care.

#### Allergies

Allergy symptoms such as a runny nose, fits of sneezing, burning and watery eyes, breathing difficulties and even breathlessness or severe itching of the skin are caused by excessive reactions of the immune system to substances (allergens) in the environment that are harmless in themselves. At a clinical level, there are diverse disease entities, for example, allergic rhinitis (hay fever), allergic bronchial asthma, atopic dermatitis, allergic contact eczema and food allergy [45]. The allergies indicator presented in this article (in contrast to reported medical diagnoses) maps the self-assessment of being currently (i.e. in the twelve months prior to the survey) affected by (any) allergic disease other than bronchial asthma. The results show that almost one-third of adult women and men in Germany are affected by allergies. Compared to the previous GEDA study (GEDA 2014/2015-EHIS), the overall 12-month prevalence of allergies has increased [46]. As expected, women (35%) are affected more frequently than men (27%). The higher prevalence of allergies observed among women with a higher level of education is also well-documented, while aspects of socialisation, awareness and use of medical services are of particular importance. A differentiated survey of individual allergic conditions would enable more specific analyses of associated factors such as age, gender and level of education. It is being discussed that for people who suffer from allergies, structural improvements to the health care system, such as a structured treatment programme for allergies (disease management programme, DMP), analogous to those for asthma and COPD, would be very helpful [45].

#### Osteoarthritis

Osteoarthritis is a degenerative disease of the joint cartilage that affects the adjacent muscles, joint capsules and ligaments [47]. Osteoarthritis is particularly widespread in the elderly population and is one of the most common diseases in old age. Compared to the results from GEDA 2014/2015-EHIS, the 12-month prevalence of osteoarthri-tis has remained fairly stable [48]. Women suffer from osteoarthritis significantly more often than men; the causes of this can be hormonal, metabolic or diet-related differences [49]. The clearly pronounced educational gradient found in women potentially indicates a connection between heavy physical labour and the development of osteoarthritis [50, 51]. The pain and loss of function associated with osteoarthritis can lead to a reduction in quality of life. Preventive measures include avoiding being overweight or overworking the joints [50, 51].

### 4.4 Strengths and limitations

The short reference period of the EHIS indicators, which were introduced to harmonise European health monitoring [13, 17], as well as the high number of cases surveyed in GEDA 2019/2020-EHIS enable the assessment of current mental and physical burdens and subjective health for adults in Germany as well as showing the patterns specific to different life stages. However, the relatively short reference period and the self-assessment of the considered EHIS indicators lead to prevalence estimates that are considerably different in some cases to those found in other health monitoring studies and epidemiological studies, which usually survey the lifetime prevalence of physician-diagnosed diseases based on medical interviews or examinations.

When comparing prevalences found in GEDA 2019/2020-EHIS and GEDA 2014/2015-EHIS, which was conducted five years earlier, the differences in study design must be taken into account, despite the largely identical operationalisation of most indicators, as they may have led to differences in the participants involved (for example, differences in the distribution of participants by education level). While GEDA 2014/2015-EHIS utilised paper and online questionnaires that were completed by each participant based on a population registry sample [2], GEDA 2019/2020-EHIS was conducted as a telephone interview survey based on a random sample of landline and mobile phone numbers [16]. Despite weighting the respective study population by age, sex, region and education level according to the composition of the population at the time of the survey – an approach discussed in more detail in relation to GEDA 2019/2020-EHIS in an article by Allen et al. in this issue of the Journal of Health Monitoring [16] – deviations in the study population with regard to other characteristics cannot be ruled out. Comparability with earlier GEDA survey waves (Infobox) conducted as telephone surveys on the basis of random samples of landline numbers is also limited as operationalisation of most indicators differ from those of the EHIS surveys. Furthermore, the GEDA 2019/2020-EHIS survey period partly coincided with the COVID-19 pandemic. The present results are based on the assumption that the sample was not systematically biased by the measures to contain the COVID-19 pandemic. Moreover, initial analyses do not reveal a systematic selection bias between the subsamples from the comparison periods April 2019 to mid-March 2020 (onset of extensive measures to contain the pandemic) and mid-March to September 2020. Nevertheless, a change in willingness to participate and its effect on the results cannot be completely ruled out. The uptake of shorter working hours and an increase in flexible working from home solutions, for example, may have made individual population groups easier (or harder) to reach by telephone.

The present study includes indicators that were selected because of their relatively high prevalence in the population and that also represent a broad spectrum of health complaints. When interpreting the results, it should be noted that the indicators occur within different time frames.

### 4.5 Conclusion

The current nationwide health monitoring data from GEDA 2019/2020-EHIS presented here demonstrate that age-related, non-communicable diseases and health-related limitations in usual everyday activities are of high public health relevance in a society faced with demographic change. A comprehensive need to provide care for health problems is nevertheless not limited to the elderly. Allergies and depressive symptoms are particularly prevalent among women and men in young and mid-adulthood, and bronchial asthma occurs with similar frequency across all age groups. Today as in the past, levels of education reflect differences in the prevalence of good subjective health, depressive symptoms, health-related limitations in everyday life and those non-communicable diseases that are among the leading causes of premature mortality, especially cardiovascular diseases, diabetes and COPD. With a knowledge of key avoidable risk factors common to these diseases, nationwide health monitoring has the task of mapping the development of risk factors and resources as well as measures to promote healthy behaviour and a healthy living environment in a timely manner. International health targets such as the sustainability goals of the United Nations 2030 Agenda can serve as a guideline here, but they must be geared toward the specific challenges of each country and region [52]. Preventive measures must above all be reviewed to determine how well they also reach disadvantaged groups such as people with lower levels of education. Health monitoring in this context has the important task of ensuring methodological comparability over time. It was not possible to conduct a regionalised analysis at the federal state level based on this initial evaluation. In future, GEDA offers the prospect of further expanding regionalised data analyses so as to enable more detailed sub-regional analyses in collaboration with the federal states. European comparisons will be possible in the future when all European data from this wave of the EHIS survey become available.

## Key statements

- Subjective health is rated less favourably with increasing age, with fewer women than men reporting their subjective health as good or very good.
- Depressive symptoms are more prevalent in young and mid-adulthood.
- The prevalences of diabetes, coronary heart disease, stroke and its sequelae as well as chronic obstructive pulmonary disease increase considerably from mid- to older adulthood and are lower or similar in women compared to men.
- The prevalence of asthma does not change with age, while the prevalence of allergies is highest in young and mid-adulthood; both of these chronic conditions are more prevalent in women than in men.
- Osteoarthritis is one of the most common chronic diseases in the elderly population and is more prevalent in women than in men.

## Figures and Tables

**Table 1:** Prevalence of subjective health rated as very good or good (n=11,953 women, n=10,681 men), of chronic diseases or long-term health problems (n=11,916 women, n=10,662 men) and long-term health-related limitations in usual everyday activities (n=11,929 women, n=10,664 men) by gender, age and education level Source: GEDA 2019/2020-EHIS

	Subjective health (very good or good)		Chronic disease or health problem (at least six months)		Health-related limitation in usual everyday activities (severe or moderate, at least six months)	
	%	(95% CI)	%	(95% CI)	%	(95% CI)
**Women (total)**	**68.6**	**(67.2–69.9)**	**51.9**	**(50.6–53.3)**	**35.5**	**(34.2–36.9)**
**Age group**							
18–29 years	87.2	(83.5–90.1)	33.8	(29.8–38.0)	16.8	(13.7–20.5)
30–44 years	82.9	(80.1–85.3)	40.9	(37.8–44.0)	21.3	(18.7–24.2)
45–64 years	66.0	(63.9–68.1)	58.6	(56.6–60.6)	39.2	(37.2–41.3)
65–79 years	55.3	(52.6–57.9)	61.9	(59.4–64.5)	46.1	(43.5–48.8)
≥80 years	42.5	(37.9–47.3)	61.9	(57.0–66.6)	63.2	(58.5–67.7)
**Education level**							
Low education group	53.5	(49.5–57.5)	56.1	(52.0–60.1)	47.3	(43.3–51.3)
Medium education group	69.1	(67.5–70.7)	53.0	(51.3–54.7)	35.3	(33.7–37.0)
High education group	82.1	(80.6–83.4)	45.2	(43.3–47.1)	24.5	(23.0–26.1)
**Men (total)**	**71.6**	**(70.2–72.9)**	**46.4**	**(44.9–47.8)**	**31.0**	**(29.7–32.4)**
**Age group**							
18–29 years	88.3	(85.2–90.8)	25.8	(22.6–29.2)	10.5	(8.4–13.1)
30–44 years	84.0	(81.2–86.4)	34.6	(31.6–37.8)	18.5	(16.0–21.3)
45–64 years	65.2	(62.8–67.5)	53.1	(50.8–55.4)	38.8	(36.5–41.3)
65–79 years	57.7	(54.6–60.8)	63.8	(60.9–66.7)	42.9	(39.9–46.0)
≥80 years	52.6	(47.1–58.0)	62.0	(56.6–67.1)	58.1	(52.6–63.4)
**Education level**							
Low education group	63.8	(58.5–68.8)	49.1	(43.8–54.4)	39.4	(34.2–44.8)
Medium education group	68.3	(66.4–70.2)	48.0	(46.0–50.1)	33.3	(31.4–35.3)
High education group	81.2	(80.0–82.3)	42.3	(40.8–43.9)	23.1	(21.9–24.5)

CI=confidence interval

**Table 2:** Prevalence of depressive symptoms in the past two weeks based on PHQ-8 by gender, age and education level (n=11,703 women, n=10,503 men) Source: GEDA 2019/2020-EHIS

	Depressive symptoms (in the past two weeks)	
	%	(95% CI)
**Women (total)**	**8.8**	**(8.0–9.7)**
**Age group** 18–29 years 30–44 years 45–64 years 65–79 years ≥80 years	11.6 8.7 10.2 5.0 7.3	(8.8–15.1) (6.8–10.9) (8.8–11.7) (3.9–6.3) (4.9–10.7)
**Education level** Low education group Medium education group High education group	13.0 8.5 5.7	(10.4–16.1) (7.4–9.6) (4.8–6.8)
**Men (total)**	**7.5**	**(6.7–8.5)**
**Age group** 18–29 years 30–44 years 45–64 years 65–79 years ≥80 Jahre	7.3 7.3 9.6 4.4 5.8	(5.3–10.0) (5.5–9.5) (8.0–11.5) (3.1–6.3) (3.8–8.7)
**Education level** Low education group Medium education group High education group	13.4 8.4 3.4	(9.9–17.9) (7.1–9.8) (2.8–4.0)

CI=confidence interval, PHQ-8=8-item Patient Health Questionnaire

**Table 3:** 12-month prevalence of diabetes (n=11,942 women, n=10,671 men), coronary heart disease (n=11,904 women, n=10,621 men) and stroke or consequences of stroke (n=11,953 women, n=10,678 men) by gender, age and education level Source: GEDA 2019/2020-EHIS

	Diabetes		Coronary heart disease		Stroke	
	%	(95% CI)	%	(95% CI)	%	(95% CI)
**Women (total)**	**8.2**	**(7.5–9.1)**	**5.1**	**(4.5–5.7)**	**2.1**	**(1.7–2.6)**
**Age group** 18–29 years 30–44 years 45–64 years 65–79 years ≥80 years	0.8^1^ 3.2 7.1 17.0 17.9	(0.2–2.6) (2.1–4.9) (6.0–8.3) (15.0–19.3) (14.4–22.0)	0.8^2^ 3.6 9.2 18.9	(0.5–1.4) (2.7–4.6) (7.8–10.9) (15.3–23.1)	0.6^2^ 1.9 3.9 5.5	(0.2–1.4) (1.3–2.7) (3.0–5.0) (3.6–8.5)
**Education level** Low education group Medium education group High education group	13.5 7.9 3.9	(11.0–16.4) (7.1–8.8) (3.4–4.5)	9.8 4.3 2.3	(7.7–12.4) (3.7–5.0) (1.9–2.8)	3.9 1.9 0.9	(2.6–5.8) (1.5–2.5) (0.7–1.2)
**Men (total)**	**9.6**	**(8.8–10.5)**	**6.6**	**(5.9–7.4)**	**2.3**	**(1.9–2.8)**
**Age group** 18–29 years 30–44 years 45–64 years 65–79 years ≥80 years	0.6 2.7 11.2 20.0 22.3	(0.3–1.2) (1.7–4.3) (9.7–13.0) (17.7–22.5) (18.1–27.2)	0.4^2^ 6.4 16.5 21.9	(0.2–0.8) (5.2–7.7) (14.2–19.1) (17.7–26.8)	0.1^1.2^ 2.4 6.2 5.9	(0.0–0.4) (1.7–3.2) (4.7–8.0) (3.9–8.8)
**Education level** Low education group Medium education group High education group	8.8 10.8 7.6	(6.2–12.3) (9.7–12.2) (6.9–8.3)	6.5 7.1 5.8	(4.3–9.6) (6.1–8.2) (5.2–6.5)	2.1 2.6 1.8	(0.9–4.7) (2.1–3.3) (1.4–2.2)

CI=confidence interval

^1^ Number of cases is n<10

^2^ Estimate refers to the age group 18–44 years

**Table 4:** 12-month prevalence of chronic obstructive pulmonary disease (n=11,940 women, n=10,665 men) and bronchial asthma (n=11,946 women, n=10,675 men) by gender, age and education level Source: GEDA 2019/2020-EHIS

	Chronic obstructive pulmonary disease		Bronchial asthma	
	%	(95% CI)	%	(95% CI)
**Women (total)**	**6.5**	**(5.8–7.2)**	**9.1**	**(8.3–9.9)**
**Age group** 18–29 years 30–44 years 45–64 years 65–79 years ≥80 years	1.2 3.9 7.7 9.0 10.9	(0.5–2.6) (2.6–5.8) (6.5–9.0) (7.7–10.6) (8.1–14.6)	7.4 8.5 10.7 8.6 7.9	(5.5–9.9) (6.9–10.6) (9.4–12.1) (7.3–10.0) (5.6–11.0)
**Education level** Low education group Medium education group High education group	9.4 6.4 3.4	(7.3–12.1) (5.6–7.3) (2.9–4.0)	10.0 9.0 8.1	(7.9–12.6) (8.1–10.1) (7.1–9.1)
**Men (total)**	**5.8**	**(5.1–6.6)**	**7.0**	**(6.3–7.7)**
**Age group** 18–29 years 30–44 years 45–64 years 65–79 years ≥80 years	1.5 2.4 7.5 10.4 9.4	(0.8–2.9) (1.5–3.7) (6.1–9.1) (8.4–12.7) (6.7–13.0)	6.5 6.7 7.2 7.4 6.7	(4.9–8.5) (5.4–8.3) (6.0–8.7) (5.9–9.1) (4.5–10.0)
**Education level** Low education group Medium education group High education group	8.6 6.4 3.5	(6.0–12.4) (5.4–7.5) (3.1–4.1)	7.3 7.4 6.1	(5.0–10.5) (6.4–8.6) (5.4–6.9)

CI=confidence interval

**Table 5:** 12-month prevalence of allergies by gender, age and education level (n=11,918 women, n=10,645 men) Source: GEDA 2019/2020-EHIS

	Allergies	
	%	(95% CI)
**Women (total)**	**34.7**	**(33.4–36.0)**
**Age group** 18–29 years 30–44 years 45–64 years 65–79 years ≥80 years	37.9 41.0 37.3 27.4 20.1	(33.8–42.1) (37.9–44.1) (35.3–39.3) (25.2–29.7) (16.5–24.2)
**Education level** Low education group Medium education group High education group	31.0 35.2 36.7	(27.4–34.8) (33.6–36.9) (34.9–38.5)
**Men (total)**	**27.0**	**(25.7–28.3)**
**Age group** 18–29 years 30–44 years 45–64 years 65–79 years ≥80 years	39.3 32.7 23.9 17.3 16.1	(35.6–43.0) (29.7–35.7) (22.0–25.9) (15.3–19.5) (12.6–20.4)
**Education level** Low education group Medium education group High education group	25.6 26.4 28.9	(21.2–30.5) (24.6–28.2) (27.4–30.4)

CI=confidence interval

**Table 6:** 12-month prevalence of osteoarthritis by gender, age and education level (n=11,859 women, n=10,630 men) Source: GEDA 2019/2020-EHIS

	Osteoarthritis	
	%	(95% CI)
**Women (total)**	**21.6**	**(20.5–22.7)**
**Age group** 18–29 years 30–44 years 45–64 years 65–79 years ≥80 years	1.1^1^ 5.1 23.9 39.7 47.3	(0.4–2.9) (3.8–6.7) (22.1–25.8) (37.2–42.4) (42.4–52.1)
**Education level** Low education group Medium education group High education group	31.2 21.0 13.5	(27.7–34.9) (19.8–22.4) (12.5–14.6)
**Men (total)**	**12.4**	**(11.5–13.4)**
**Age group** 18–29 years 30–44 years 45–64 years 65–79 years ≥80 years	1.1^1^ 3.5 15.4 23.2 31.6	(0.5–2.6) (2.5–5.1) (13.8–17.2) (20.7–26.0) (26.7–36.9)
**Education level** Low education group Medium education group High education group	13.3 12.8 11.4	(10.0–17.4) (11.5–14.1) (10.5–12.3)

CI=confidence interval

^1^ Number of cases is n<10

## Appendix

**Annex Table 1:** Prevalence of subjective health rated as very good or good (n=11,953 women, n=10,681 men), chronic diseases or long-term health problems (n=11,916 women, n=10,662 men) and long-term health-related limitations in usual everyday activities (n=11,929 women, n=10,664 men) by gender, age and education level Source: GEDA 2019/2020-EHIS

	Subjective health (very good or good)		Chironic disease or health problem (at least six months)		Health-related limitation in usual everyday activities (severe or moderate, at least six months)	
	%	(95% CI)	%	(95% CI)	%	(95% CI)
**Women (total)**	**68.6**	**(67.2–69.9)**	**51.9**	**(50.6–53.3)**	**35.5**	**(34.2–36.9)**
**Age group and education level**						
**18–29 years**	87.2	(83.5–90.1)	33.8	(29.8–38.0)	16.8	(13.7–20.5)
Low education group	78.1	(67.1–86.2)	36.8	(26.8–48.0)	25.4	(16.6–36.9)
Medium education group	87.4	(82.4–91.1)	33.9	(28.7–39.6)	16.3	(12.5–21.0)
High education group	95.0	(91.4–97.1)	31.3	(24.9–38.4)	10.6	(7.0–15.5)
**30–44 years**	82.9	(80.1–85.3)	40.9	(37.8–44.0)	21.3	(18.7–24.2)
Low education group	74.1	(61.8–83.5)	34.4	(23.5–47.3)	25.5	(16.3–37.6)
Medium education group	81.0	(77.1–84.4)	44.7	(40.4–49.1)	23.3	(19.7–27.4)
High education group	89.6	(86.5–92.1)	37.6	(34.0–41.3)	16.4	(13.6–19.7)
**45–64 years**	66.0	(63.9–68.1)	58.6	(56.6–60.6)	39.2	(37.2–41.3)
Low education group	50.4	(42.8–58.0)	67.4	(59.6–74.4)	49.3	(41.6–56.9)
Medium education group	65.5	(63.0–68.0)	59.6	(57.1–62.0)	40.7	(38.3–43.3)
High education group	78.6	(76.5–80.6)	49.0	(46.6–51.5)	27.7	(25.5–29.9)
**65–79 years**	55.3	(52.6–57.9)	61.9	(59.4–64.5)	46.1	(43.5–48.8)
Low education group	46.1	(39.2–53.1)	62.4	(55.3–69.0)	52.4	(45.3–59.3)
Medium education group	56.4	(53.4–59.3)	61.8	(58.9–64.6)	44.5	(41.6–47.5)
High education group	67.6	(64.6–70.5)	62.1	(59.0–65.0)	40.8	(37.8–44.0)
**≥80 years**	42.5	(37.9–47.3)	61.9	(57.0–66.6)	63.2	(58.5–67.7)
Low education group	37.9	(29.9–46.7)	61.1	(52.2–69.4)	66.5	(57.9–74.1)
Medium education group	46.1	(40.8–51.4)	62.3	(56.8–67.4)	60.3	(54.9–65.4)
High education group	53.4	(46.5–60.0)	65.1	(58.4–71.4)	58.5	(51.6–65.0)
**Men (total)**	**71.6**	**(70.2–72.9)**	**46.4**	**(44.9–47.8)**	**31.0**	**(29.7–32.4)**
**Age group and education level**						
**18–29 years**	88.3	(85.2–90.8)	25.8	(22.6–29.2)	10.5	(8.4–13.1)
Low education group	80.9	(72.4–87.3)	31.8	(24.4–40.3)	14.8	(9.6–22.2)
Medium education group	89.8	(85.8–92.8)	23.5	(19.5–28.0)	9.8	(7.2–13.1)
High education group	94.4	(90.8–96.6)	25.0	(20.0–30.7)	7.4	(4.7–11.5)
**30–44 years**	84.0	(81.2–86.4)	34.6	(31.6–37.8)	18.5	(16.0–21.3)
Low education group	74.3	(61.8–83.8)	32.7	(22.0–45.5)	25.1	(15.7–37.5)
Medium education group	80.4	(76.4–83.9)	39.1	(34.6–43.8)	22.1	(18.5–26.3)
High education group	92.8	(90.6–94.5)	29.5	(26.2–33.1)	10.9	(8.7–13.6)
**45–64 years**	65.2	(62.8–67.5)	53.1	(50.8–55.4)	38.8	(36.5–41.3)
Low education group	47.4	(37.4–57.6)	66.8	(56.5–75.7)	61.9	(51.5–71.3)
Medium education group	60.1	(56.8–63.4)	55.8	(52.5–59.0)	42.8	(39.5–46.2)
High education group	80.4	(78.4–82.2)	43.6	(41.2–46.0)	23.6	(21.7–25.7)
**65–79 years**	57.7	(54.6–60.8)	63.8	(60.9–66.7)	42.9	(39.9–46.0)
Low education group	48.5	(34.3–62.9)	67.4	(52.0–79.8)	55.0	(40.3–68.9)
Medium education group	53.1	(48.7–57.5)	65.8	(61.5–69.8)	45.0	(40.7–49.4)
High education group	68.1	(65.3–70.7)	59.9	(57.1–62.6)	36.3	(33.6–39.0)
**≥80 years**	52.6	(47.1–58.0)	62.0	(56.6–67.1)	58.1	(52.6–63.4)
Low education group	54.5	(34.9–72.8)	67.0	(46.4–82.7)	63.7	(43.1–80.2)
Medium education group	48.0	(40.5–55.5)	62.1	(54.5–69.1)	58.8	(51.2–66.0)
High education group	58.6	(53.2–63.8)	60.4	(55.0–65.6)	54.2	(48.9–59.5)

CI=confidence interval

**Annex Table 2:** Prevalence of depressive symptoms in the past two weeks based on PHQ-8 by gender, age and education level (n=11,703 women, n=10,503 men) Source: GEDA 2019/2020-EHIS

	%	(95% CI)		%	(95% CI)
Women (total)	8.8	(8.0–9.7)	Men (total)	7.5	(6.7–8.5)
**Age group and education level**			**Age group and education level**		
**18–29 years** Low education group Medium education group High education group	11.6 24.0 10.3 4.4	(8.8–15.1) (15.2–35.6) (7.1–14.6) (2.2–8.5)	**18–29 years** Low education group Medium education group High education group	7.3 12.5 6.6 3.0	(5.3–10.0) (7.4–20.4) (4.2–10.1) (1.6–5.3)
**30–44 years** Low education group Medium education group High education group	8.7 13.6 9.4 5.4	(6.8–10.9) (7.1–24.4) (6.9–12.7) (3.7–8.0)	**30–44 years** Low education group Medium education group High education group	7.3 11.9^1^ 9.2 2.9	(5.5–9.5) (5.6–23.4) (6.6–12.6) (1.9–4.5)
**45–64 years** Low education group Medium education group High education group	10.2 18.0 9.5 6.6	(8.8–11.7) (12.8–24.7) (7.9–11.3) (5.4–8.1)	**45–64 years** Low education group Medium education group High education group	9.6 18.6 11.3 3.6	(8.0–11.5) (11.5–28.6) (9.0–14.1) (2.7–4.6)
**65–79 years** Low education group Medium education group High education group	5.0 6.0 4.5 5.3	(3.9–6.3) (3.4–10.5) (3.4–5.9) (3.9–7.1)	**65–79 years** Low education group Medium education group High education group	4.4 11.9^1^ 3.9 2.7	(3.1–6.3) (4.4–28.3) (2.6–5.8) (1.9–3.8)
**≥80 years** Low education group Medium education group High education group	7.3 7.9 7.1 5.9	(4.9–10.7) (3.9–15.4) (4.8–10.4) (3.4–10.1)	**≥80 years** Low education group Medium education group High education group	5.8 5.0^1^ 5.5 7.0	(3.8–8.7) (0.9–24.3) (3.0–9.7) (4.5–10.8)

CI=confidence interval, PHQ-8=8-item Patient Health Questionnaire

^1^ Number of cases is n<10
